# Epac1 contributes to apremilast-mediated rescue of pemphigus autoantibody-induced loss of keratinocyte adhesion

**DOI:** 10.1172/jci.insight.187481

**Published:** 2025-04-29

**Authors:** Anna M. Sigmund, Franziska C. Bayerbach, Daniela Kugelmann, Elisabeth Butz, Sina Moztarzadeh, Margarethe E.C. Schikora, Anja K.E. Horn, Mariya Y. Radeva, Sophia Engelmayer, Desalegn T. Egu, Matthias Goebeler, Enno Schmidt, Jens Waschke, Franziska Vielmuth

**Affiliations:** 1 Chair of Vegetative Anatomy, Institute of Anatomy, Faculty of Medicine, LMU Munich, Munich, Germany.; 2 Department of Dermatology, Venereology and Allergology, University Hospital Würzburg, Würzburg, Germany.; 3 Institute of Experimental Dermatology, University of Lübeck, Lübeck, Germany.; 4 Department of Dermatology, University Clinic of Schleswig-Holstein, Campus Lübeck, Lübeck, Germany.

**Keywords:** Cell biology, Dermatology, Autoimmune diseases, Cell migration/adhesion, Skin

## Abstract

In the bullous autoimmune disease pemphigus vulgaris (PV), autoantibodies directed mainly against desmoglein 1 (Dsg1) and Dsg3 cause loss of desmosomal adhesion. We recently showed that intracellular cAMP increase by the phosphodiesterase 4 inhibitor apremilast was protective in different PV models. Thus, we here analyzed the involvement of the cAMP effector exchange factor directly activated by cAMP1 (Epac1). In Epac1-deficient mice pemphigus antibody-induced blistering was ameliorated in vivo while apremilast had no additional effect. Interestingly, augmented protein levels of Dsg1 and Dsg3 as well as increased Dsg1 mRNA levels and higher numbers of Dsg1- and Dsg3-dependent single-molecule interactions were detected in keratinocytes derived from Epac1-deficient mice. This was paralleled by stronger intercellular adhesion under baseline conditions and prevention of pemphigus autoantibody-induced loss of intercellular adhesion. However, the protective effect of apremilast against loss of intercellular adhesion in response to the pathogenic Dsg3 antibody AK23 was attenuated in Epac1-deficient keratinocytes. Similarly, the Epac1 inhibitor Esi09 protected keratinocytes from pemphigus antibody-induced loss of adhesion. Mechanistically, Epac1 deficiency resulted in lack of apremilast-induced Rap1 activation and phosphorylation of Pg at S665. Taken together, these data indicate that Epac1 is involved in the regulation of baseline and cAMP-mediated stabilization of keratinocyte adhesion.

## Abstract 2

Exchange factor directly activated by cAMP 1 is involved in regulation of adhesion in keratinocytes and in protective cAMP signaling in pemphigus.

## Introduction

Desmosomes are multiprotein complexes, mediating strong intercellular adhesion and thereby providing epidermal integrity (1). They consist of desmosomal cadherins of the desmoglein (Dsg) and desmocollin (Dsc) subfamilies, which maintain strong adhesion by intercellular homo- and heterophilic interactions and plaque proteins such as plakoglobin (Pg) and desmoplakin (Dp), which are linked to the keratin cytoskeleton (2, 3).

The importance of desmosomal adhesion is reflected in the life-threatening bullous autoimmune disease pemphigus vulgaris (PV). In pemphigus, autoantibodies (PV-IgGs) directed against Dsg1 and Dsg3, but also against other desmosomal and nondesmosomal proteins, cause flaccid blistering of the epidermis and mucous membranes (4, 5). Binding of PV-IgGs directly inhibits interactions of desmosomal cadherins (6–8) but also induces a plethora of signaling pathways such as p38MAPK, PLC, ERK, and Src-EGFR (9–14). Autoantibody-binding finally results in depletion of desmogleins from the cell membrane as well as retraction of the keratin cytoskeleton from the desmosomal plaque (15).

Up to now, pemphigus therapies mainly focus on suppression or modulation of the immune system, which may result in severe adverse events (16–18). Thus, new treatment paradigms stabilizing keratinocyte cohesion in the presence of autoantibodies would fulfill an unmet medical need, especially in the acute phase of the disease.

We recently reported that intracellular cAMP elevation induces a PKA-dependent phosphorylation of Pg at serine (S) 665 in both keratinocytes and cardiomyocytes, which is paralleled by improved keratin association to the desmosomal plaque in keratinocytes (19, 20). cAMP increase mediated by the phosphodiesterase 4 (PDE4) inhibitor apremilast was protective against PV-IgG–induced loss of intercellular adhesion in vitro in human keratinocytes, ex vivo in human skin, and in vivo in mice (19). Importantly, apremilast is approved for treatment of other skin diseases, such as psoriasis, and was already used off label to treat patients with therapy-resistant PV (21–26). Therefore, understanding the mechanisms of apremilast-induced stabilization of keratinocyte cohesion would be of high clinical relevance not only for approval of apremilast but also for establishing other new treatment approaches.

The nucleotide exchange factor for the Rap family of small GTPases, exchange factor directly activated by cAMP1 (Epac1), is, besides PKA, the main cAMP-effector protein (27, 28). Among many other cellular functions, Epac1 is involved in integrin- but also in E-Cadherin–mediated adhesion and desmosome assembly (29, 30).

In the current study, we aim to unravel the impact of Epac1 on the mechanism of apremilast-mediated rescue from PV-IgG–induced loss of adhesion. We show that Epac1 contributes to apremilast effects and this effect is probably dependent on Rap1 activation and phosphorylation of Pg at S665.

## Results

### Epidermis of Epac1-ko mice shows no alterations.

**Figure 1:**
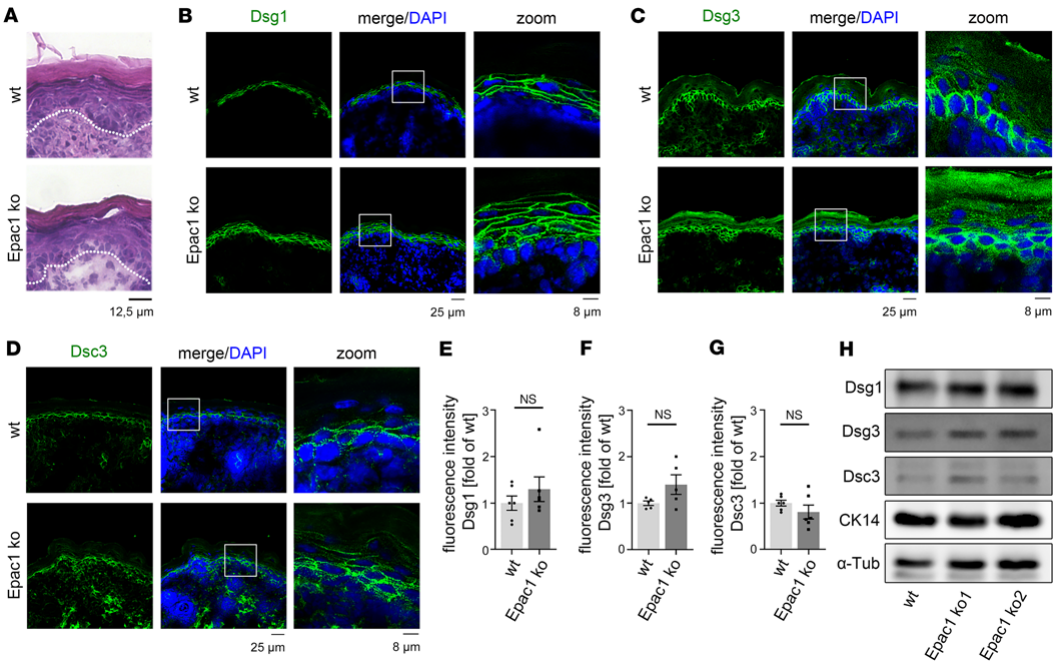
Epidermis of Epac1-ko mice shows no alterations. (**A**) H&E staining of murine epidermis shows no differences between WT and Epac1-ko mice. *n* = 4. (**B**–**D**) Immunostaining for Dsg1, Dsg3, and Dsc3 shows no difference in the expression pattern of WT and Epac1-ko mice (representative of *n* > 5). (**E**–**G**) Quantification of epidermal staining of Dsg1, Dsg3, and Dsc3. (**H**) Western blot analysis of epidermis obtained from WT and 2 different Epac1-ko mice shows no significant alterations for desmosomal proteins (representative of *n* > 3). Columns indicate mean value ± SEM, *P < 0.05. 2-tailed Student’s *t* test. α-Tub, α-Tubulin, CK14, Cytokeratin 14. Scale bars: 12.5 μm (**A**); 25 μm (**B**, **C**, and **D**, left and middle); 8 μm (**B**, **C**, and **D**, right).

To analyze the role of Epac1 in apremilast-mediated signaling, we studied the skin of Epac1-ko mice. H&E staining of the epidermis revealed normal morphology and no alterations in thickness or layer formation (Figure 1A and Supplemental Figure 1A; supplemental material available online with this article; https://doi.org/10.1172/jci.insight.187481DS1). We next performed immunostaining to analyze a potential effect of Epac1 on the expression pattern of several desmosomal proteins (Figure 1, B–D). In WT mice, Dsg1 was expressed primarily in superficial layers, and no significant difference with regard to intensity and expression pattern was detected in the epidermis from Epac1-ko mice (Figure 1, B and E). Similarly, no significant changes in the expression patterns of Dsg3, desmocollin 3 (Dsc3) (Figure 1, C, D, F, and G), and Dsc1 (Supplemental Figure 1, B and C) were detected between WT and Epac1 epidermis. Dsc3 and Dsg3 were abundant mainly in basal layers, while Dsc1 was mainly expressed in superficial layers, as expected (1). Western blot analysis of whole skin lysates confirmed these results. Protein expression of all analyzed desmogleins and desmocollins as well as cytokeratin 14 (CK14) did not differ significantly between Epac1-ko and WT mice (Figure 1H and Supplemental Figure 1, D–G).

### Desmosomal protein levels are increased in murine Epac1-ko keratinocytes.

**Figure 2:**
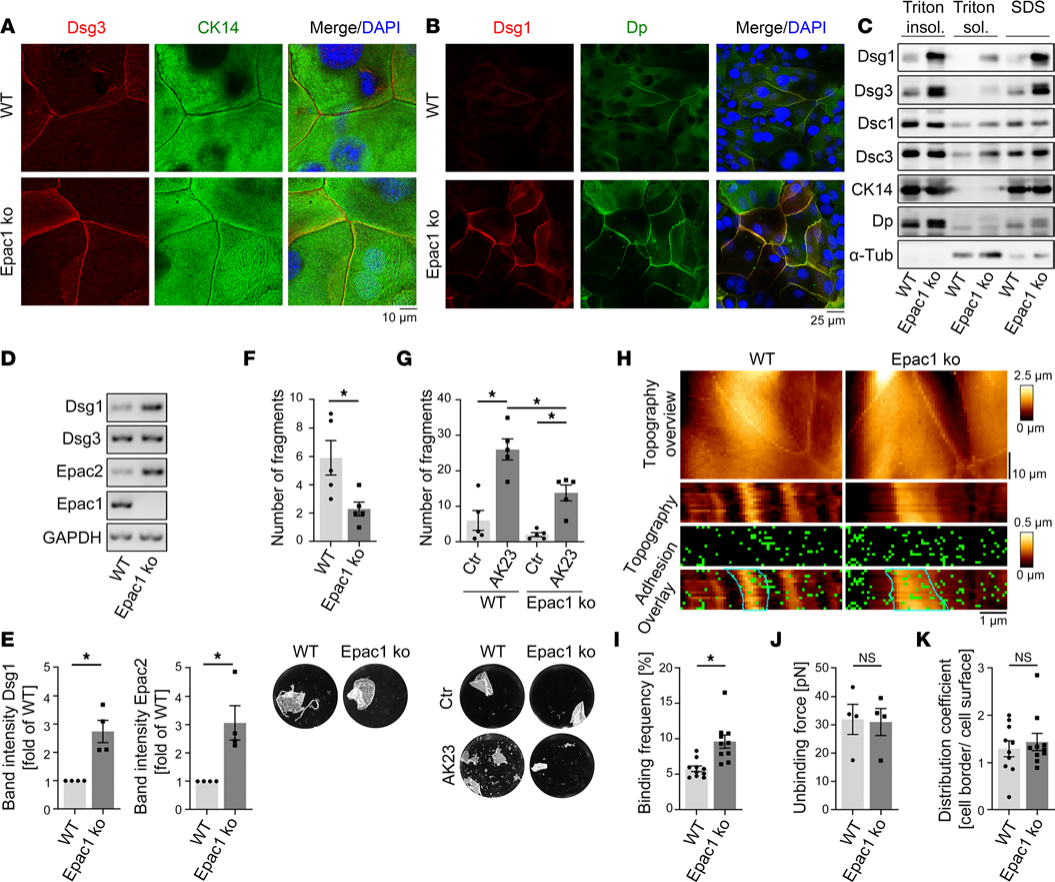
Murine Epac1-ko keratinocytes display upregulated desmosomal proteins and strengthened intercellular adhesion. Costaining of Dsg3/CK14 (**A**) and Dsg1/Dp (**B**) in murine keratinocytes show upregulation of Dsg1 and Dsg3 in Epac1-ko cells (Representative of *n* > 4). (**C**) Western blot analysis of Triton fractionation and whole SDS cell lysates of murine keratinocytes shows upregulation of Dsg1 and Dsg3 (representative of *n* > 3). (**D**) PCR analysis of WT and Epac1-ko mRNA displays upregulation of Dsg1 and Epac2 mRNA due to missing Epac1 (representative of *n* = 4). (**E**) Quantification of data presented in **D**. Keratinocyte dissociation assays under basal conditions (**F**) and after treatment with AK23 (**G**) (representative of *n* > 4). Adhesion of Epac1-ko keratinocytes is strengthened compared with WT cells. (**H**) Topography overview images of AFM measurements on living keratinocytes using Dsg1-functionalized tips reveal heightened cell borders. Small areas along the cell borders were chosen for adhesion measurements. Each pixel represents a force-distance curve. In the adhesion panel, each green pixel represents a Dsg1-specific binding event. Cell borders are marked as blue dotted line. (**I**–**K**) Quantification of AFM adhesion measurements. Epac1-ko cells displayed higher binding frequencies (**I**) compared with WT cells, whereas unbinding forces (**J**) and distribution of binding events (**K**) were unaltered. (10 cell borders from 4–5 independent experiments with 900 force-distance curves per cell border). Bars indicate mean value ± SEM. *P < 0.05. 2-tailed Student’s *t* test (**E**, **F**, and **I**–**K**), 2-way ANOVA with Bonferroni correction (**G**). α-Tub, α-Tubulin;CK14, cytokeratin 14; Dp, desmoplakin. Scale bars: 10 μm (**A**); 25 μm (**B**); 10 μm (**H** top) 1 μm (**H** bottom).

To further investigate the role of Epac1, we generated murine keratinocyte cell lines from Epac1-ko and corresponding WT mice. Immunostaining of keratins revealed a normal, dense, and regular mesh in both cell lines, and slightly increased staining intensity was observed in Epac1-ko cells (Figure 2A). In contrast, Epac1-ko keratinocytes showed enhanced Dsg1 and Dsg3 staining at the cell borders compared with WT cells (Figure 2, A and B, and Supplemental Figure 2A). In line, Western blot analysis of Epac1-ko keratinocytes showed increased protein levels of Dsg1 and Dsg3 in the desmosome-containing Triton-insoluble fraction as well as the whole cell lysates (Figure 2C and Supplemental Figure 2, B and C). Levels of Dsc1 and Dsc3 were comparable in ko keratinocytes and WT cells (Figure 2C, Supplemental Figure 2D, E). Interestingly, however, PCR analysis revealed increased mRNA levels for Dsg1 but not for Dsg3 in Epac1-ko keratinocytes when compared with WT cells (Figure 2, D and E, and Supplemental Figure 2F).

We previously described a compensatory upregulation of Epac2 in Epac1-deficient endothelial cells (31). Similarly, reverse PCR analysis demonstrated upregulation of Epac2 mRNA in Epac1-ko cells compared with WT cells (Figure 2, D and E).

Next, we asked whether higher levels of desmosomal proteins in Epac1-deficient keratinocytes influence intercellular adhesion and thus performed a keratinocyte dissociation assay. Indeed, absence of Epac1 resulted in better intercellular adhesion in untreated keratinocytes (Figure 2F). Moreover, loss of adhesion, induced by the pathogenic anti-Dsg3 antibody AK23 was attenuated in Epac1-ko cells (Figure 2G).

To analyze the effect of Epac1 deficiency on Dsg1 single molecule binding properties, we performed atomic force microscopy (AFM) adhesion measurements with a Dsg1-functionalized tip on living keratinocytes (Figure 2, H–K). Topography and Dsg1 unbinding forces conformed previous studies and showed no differences (32, 33) (Figure 2, H and J). In line with higher Dsg1 levels and strengthened adhesion of Epac1-ko keratinocytes, Dsg1 binding frequencies were elevated in these cells (Figure 2, H and I). Similar results were obtained for Dsg3 binding properties (Supplemental Figure 2, G–I). In contrast to distribution of Dsg1-dependent binding events, which did not differ between WT and ko cells, Epac1-ko cells showed more Dsg3-dependent binding events at the cell borders (Figure 2K and Supplemental Figure 2J). Taken together, these results indicate a compensatory upregulation of desmosomal proteins and improved intercellular baseline adhesion in Epac1-deficient keratinocytes.

### The protective effect of apremilast but not of forskolin/rolipram on PV-autoantibody–induced loss of adhesion is dependent on Epac1.

**Figure 3:**
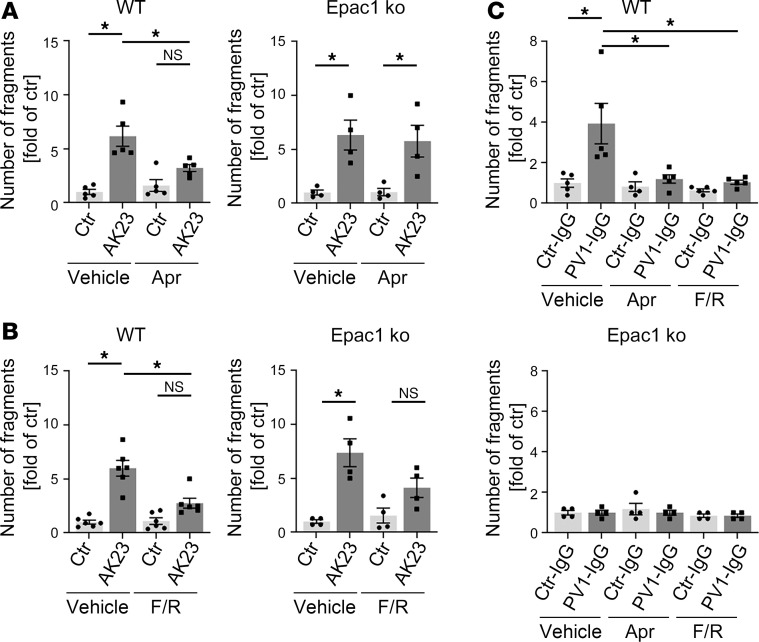
Protective effect of apremilast is ameliorated in Epac1-ko keratinocytes. Keratinocyte dissociation assays in WT or Epac1-ko keratinocytes after apremilast (Apr) (**A**) or forskolin/rolipram (F/R) (**B**) treatment followed by AK23 incubation (*n* > 4). (**C**) Epac1-ko keratinocytes, show, in contrast to WT cells, no loss of adhesion upon PV1-IgG treatment (*n* > 4). Bars indicate mean value ± SEM. *P < 0.05. 2-way ANOVA with Bonferroni correction. PV-IgG, Pemphigus vulgaris IgG; ctr-IgG, IgG of healthy volunteers.

Recently, we observed a protective effect of increased intracellular cAMP by the PDE4 inhibitor apremilast on PV-induced loss of adhesion. This was paralleled by a PKA-induced phosphorylation of Pg at S665 (19). However, the role of the other central cAMP downstream signaling molecule Epac1 was not analyzed in this study. Thus, we treated WT and Epac1-ko keratinocytes with apremilast followed by application of the pathogenic anti-Dsg3 antibody, AK23, and performed keratinocyte dissociation assays. Surprisingly, pretreatment with apremilast protected WT but not Epac1 -ko keratinocytes from AK23-induced loss of adhesion, demonstrating that Epac1 is required for cAMP-mediated protective effects on keratinocyte adhesion (Figure 3A and Supplemental Figure 3A). cAMP ELISA upon apremilast treatment revealed a significant increase in WT as well as Epac1-ko cells, demonstrating that apremilast was effective in the enhancement of cAMP levels in both cell lines, and this was independent of loss of Epac1 (Supplemental Figure 3B). In contrast, higher cAMP levels in response to treatment with the combination of the adenylate cyclase activator forskolin and the **PDE4 inhibitor** rolipram ameliorated AK23-induced loss of adhesion in WT as well as in Epac1-ko cells (Figure 3B and Supplemental Figure 3, C and D), indicating that different downstream molecules are involved in protective cAMP signaling in pemphigus.

Presumably because of higher baseline adhesion in Epac1-ko keratinocytes, IgG from a patient with pemphigus vulgaris (PV1-IgG) induced loss of adhesion in WT but not in Epac1-deficient cells (Figure 3C). The latter observation was confirmed, for both cell lines, using PV2-IgG from a different patient with PV (Supplemental Figure 3E). Next, we used the Epac1-specific inhibitor Esi-09 in a keratinocyte dissociation assay. Similar to Epac1 ko, Esi-09 treatment in WT cells strengthened cell contacts and prevented AK23-induced loss of adhesion, whereas apremilast had no additional effect (Supplemental Figure 3, F and G).

**Figure 4:**
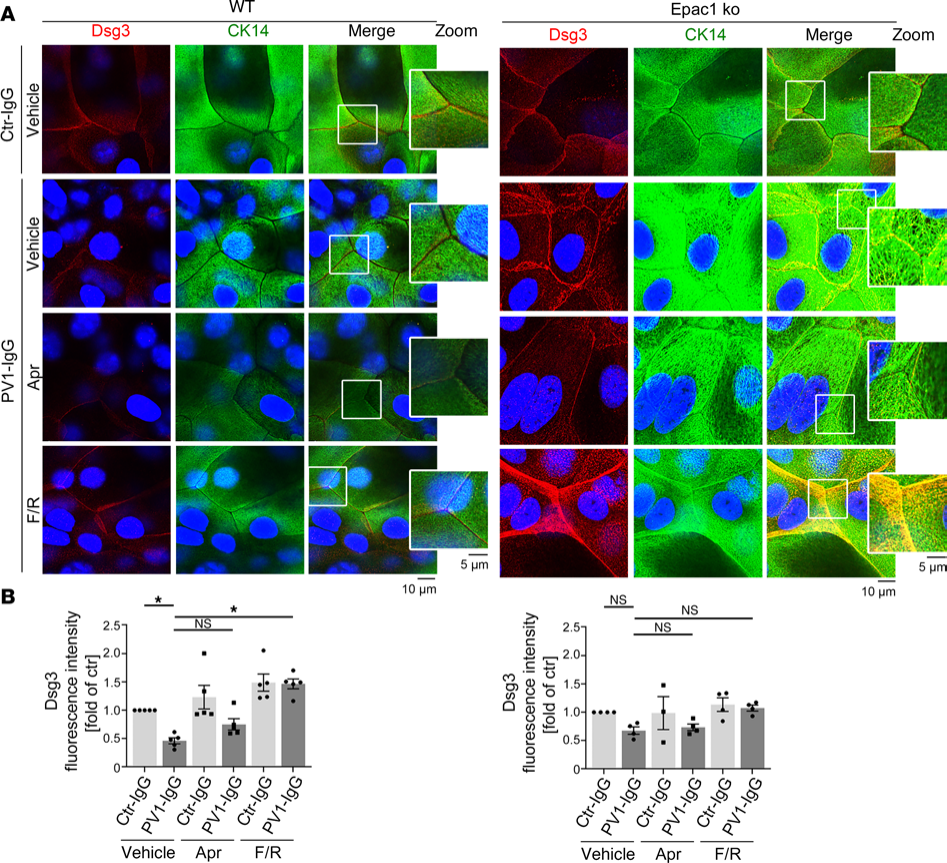
Apremilast rescues PV-IgG-induced alteration of keratins Epac1 dependent. (**A**) Immunofluorescence of Dsg3/CK14 after pretreatment with either apremilast (Apr) or forskolin/rolipram (F/R). PV1-IgG-induced fragmentation of Dsg3 staining in both cell lines is rescued upon F/R only (representative of *n* > 3). The protective effect of Apr on keratin alterations after PV1-IgG treatment is diminished in Epac1-ko cells. DAPI (blue) was added to stain nuclei. (**B**) Quantification of Dsg3 fluorescence intensities. Bars indicate mean value ± SEM. *P < 0.05. 2-way ANOVA with Bonferroni correction. Pemphigus vulgaris IgG (PV-IgG), IgG of healthy volunteers (ctr-IgG), cytokeratin 14 (CK14). Scale bars: 10 μm; 5 μm (insets).

To analyze the effect of PV-IgG in Epac1 ko, we treated cells with control-IgG (ctr-IgG) obtained from healthy human volunteers or PV1-IgG followed by vehicle control, apremilast, or F/R administration and performed immunofluorescence staining for Dsg3 and CK14 (Figure 4A). Under control conditions, staining for Dsg3 was linear along cell borders, and keratin staining revealed a dense and regular network in the cytoplasm of WT and Epac1-ko keratinocytes (Figure 4A). In contrast, PV1-IgG treatment induced fragmentation and reduction of Dsg3 staining in WT, and mild alterations in Epac1-ko, cells (Figure 4, A and B). Additionally, in both cell lines, PV1-IgG treatment induced an irregular and broadened keratin-mesh formation, thickened keratin bundles, and retraction of the keratins from the cell periphery, as described before (33). In line with our previous studies, apremilast ameliorated PV1-IgG–induced alterations of the keratin network but not depletion of Dsg3 staining in WT cells, whereas F/R did improve keratin as well as Dsg3 staining (Figure 4A) (19, 34). In accordance with the data of the keratinocyte dissociation assays, apremilast did not improve any PV1-IgG–induced effects in Epac1-ko cells. In contrast, F/R rescued Epac1-ko keratinocytes from PV1-IgG–induced keratin alterations and Dsg3 depletion (Figure 4B).

Taken together, lower cAMP levels in response to apremilast ameliorated PV-IgG–induced loss of adhesion by strengthening the keratin network in an Epac1-dependent manner. In contrast, the combination of forskolin and rolipram rescued both PV-IgG–induced keratin cytoskeleton alterations as well as Dsg3 depletion, most likely due to higher levels of cAMP.

### Apremilast induces cAMP downstream signaling dependent on Epac1.

**Figure 5:**
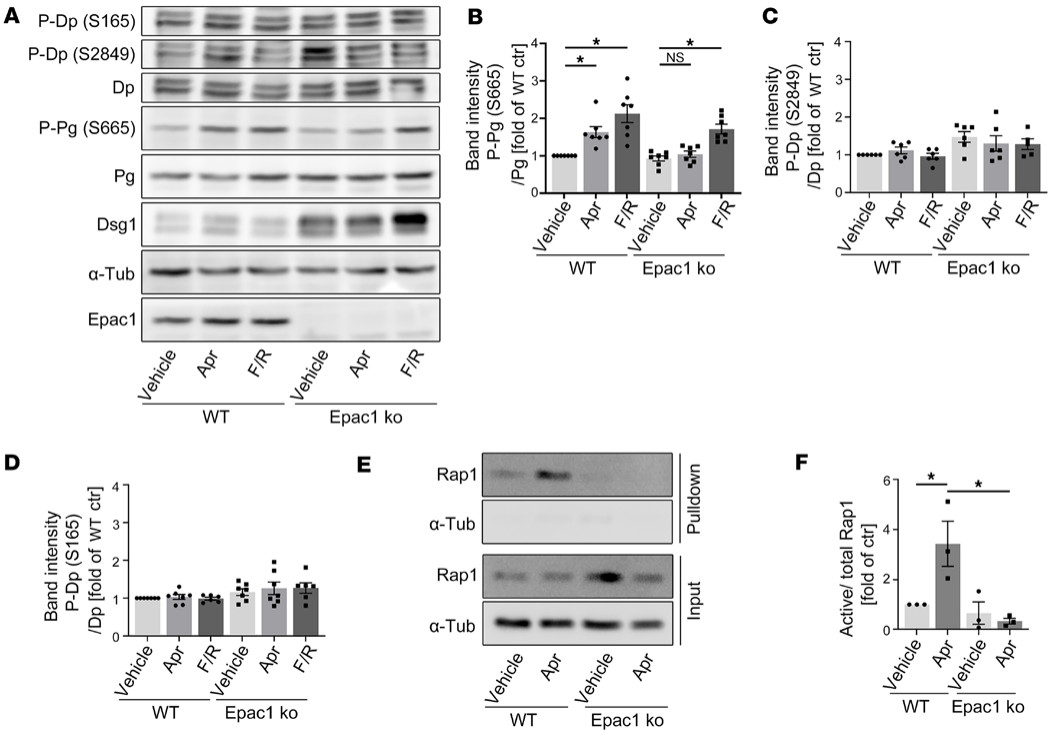
Apremilast induces phosphorylation of Pg and Rap1 activation in a Epac1-dependent manner. (**A**) Western blot analysis of WT and Epac1-ko keratinocyte lysates after treatment with apremilast (Apr) or forskolin/rolipram (F/R) for 1 hour (representative of *n* > 6). Phosphorylation status of desmoplakin (Dp) and plakoglobin (Pg) were analyzed. Epac1 ko was verified and α-tubulin (α-tub) was used as a loading control. Pg is phosphorylated upon F/R treatment independently of Epac1 and upon Apr treatment in WT cells only. Neither phosphorylation on S165 nor S2849 of Dp was affected by Apr or F/R. Quantification of P-Pg (S665) (**B**), P-Dp (S2849) (**C**),and P-Dp (S165) (**D**). (**E**) Active Rap1 upon Apr treatment in WT and Epac1-ko keratinocytes was pulled down and analyzed by Western blot (representative of *n* = 3). (**F**) Quantification of Rap1 activation. Bars indicate mean value ± SEM. *P < 0.05, 2-way ANOVA with Bonferroni (**B**–**D**) or Tukey (**F**) correction.

Next, we analyzed potential Epac1 downstream targets that may be responsible for strengthening of keratinocyte adhesion upon apremilast treatment. Coupling of keratins to the desmosomal plaque was shown to be regulated positively by a PKA-mediated phosphorylation of Pg at S665 and negatively by phosphorylation of Dp at S2849 by PKC (28, 35). We showed recently that the protective effect of apremilast in PV was paralleled by a PKA-dependent phosphorylation of Pg on S665 (19). Western blot analysis of WT keratinocytes confirmed phosphorylation of Pg at S665 by apremilast as well as by F/R (Figure 5, A and B). Surprisingly, and in contrast to F/R-induced phosphorylation, apremilast-induced phosphorylation was absent in Epac1-deficient cells, suggesting that Epac1 appears to be required for PKA-dependent Pg-phosphorylation at S655A, due to apremilast treatment. In contrast, phosphorylation of Dp at both S165 and S2849 was changed neither in WT nor in Epac1-ko keratinocytes upon treatment with apremilast or F/R (Figure 5, A, C, and D).

Since Epac1 serves as a guanine nucleotide–exchange factor for the small GTPases Rap1 and Rap2 (36), we performed Rap1 pull-down assays to analyze Rap1 activation upon apremilast treatment in WT and Epac1-ko cells. Apremilast induced activation of Rap1 in WT but not in Epac1-ko keratinocytes (Figure 5, E and F).

### Epac1 deficiency attenuates pemphigus autoantibody-induced blistering and the protective effect of apremilast in the pemphigus mouse model in vivo.

**Figure 6:**
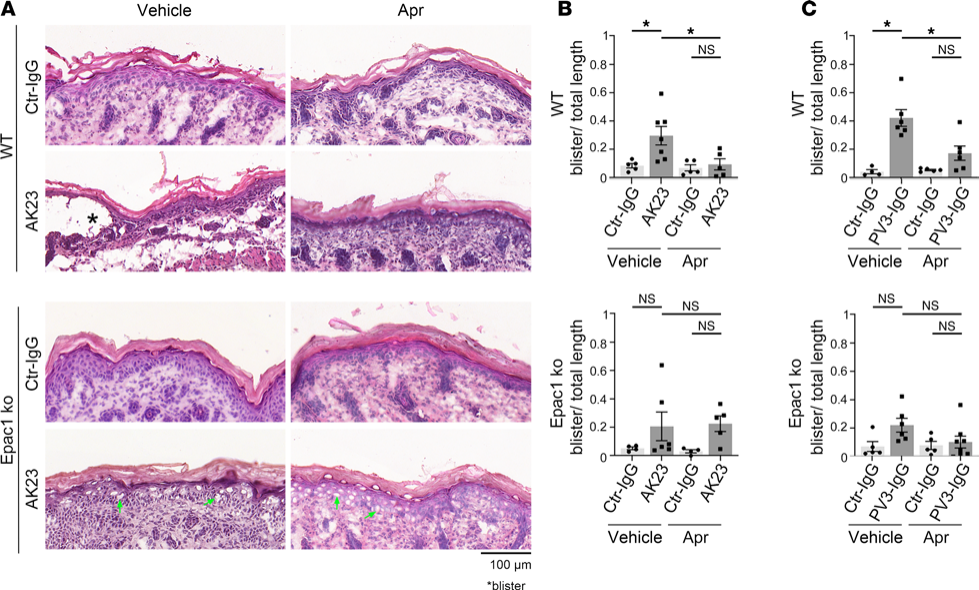
Epac1 depletion attenuates blistering in the pemphigus mouse model and abrogates the protective effect of apremilast. (**A**) H&E staining of neonatal mouse skin after injection of vehicle or apremilast (Apr) prior to injection of AK23 or ctr-IgG (representative of *n* > 4). AK23 induced suprabasal blistering of WT epidermis, which was rescued by apremilast. Epac1-ko epidermis showed only microblisters (marked with green arrows) after AK23 injection, which were not improved by apremilast. (**B**) Quantification of blistered epidermis shows a protective effect of apremilast in WT but not in Epac1-ko mice. (**C**) Quantification of blistered epidermis after PV3-IgG injection shows a protective effect of apremilast in WT but not in Epac1-ko mice (representative of *n* > 4). Columns indicate mean value ± SEM, *P < 0.05. 2-way ANOVA with Bonferroni correction. PV-IgG, Pemphigus vulgaris IgG, ctr-IgG, IgG of healthy volunteers. Scale bars: 100 μm.

To analyze the effects of Epac1 deficiency on the protective effect of apremilast in vivo we used a neonatal pemphigus mouse model. WT or Epac1-ko mice were injected with vehicle or apremilast 2 hours prior to AK23 injection. After 10 hours, mice were sacrificed and skin was harvested for further histological analysis. AK23 induced significant blistering of WT epidermis (Figure 6, A and B) while only mild effects of AK23 were observed in Epac1-ko epidermis. Preinjection of apremilast prevented blister formation in WT mice, and, in line with the keratinocyte dissociation assay, it was not effective to prevent microblisters in Epac1-ko mice. Repeating the experiment with PV3-IgG revealed comparable results in WT animals. However, similar to the results in the keratinocyte dissociation assay, Epac1-ko epidermis appeared to be more stable, since PV3-IgG did induce small blisters only and displayed no significant effect of apremilast (Figure 6C and Supplemental Figure 4). Taken together, in vivo animal experiments revealed a better adhesion of Epac1-ko keratinocytes, and an Epac1-dependent protective effect of apremilast on pemphigus antibody induced blister formation, validating the in vitro results.

## Discussion

Pemphigus therapies are limited to suppression or modulation of the immune system and may be associated with adverse side effects. Activation of cAMP signaling by the PDE4 inhibitor apremilast represents a promising strategy to strengthen desmosomal adhesion and thereby prevent acantholysis in PV (19, 21, 23, 34). Thus, it is important to unravel its mechanism of action. Here, we report an important role for the Rap1 guanine nucleotide–exchange factor Epac1 for mediating the downstream effects of an apremilast-induced cAMP increase. The protective effect of apremilast against the pathogenic Dsg3 antibody AK23 was dependent on Epac1 in vitro and in vivo. Mechanistically, Epac1 was important for apremilast-induced activation of Rap1 and phosphorylation of Pg at S665.

### Compensatory upregulation of desmosomal proteins in Epac1-ko keratinocytes strengthens desmosomal adhesion.

To understand cAMP-induced strengthening of keratinocyte adhesion, we analyzed the Rap1 GEF and cAMP effector protein Epac1. We observed no alterations in Epac1-ko mouse epidermis with respect to thickness and desmosomal protein levels. Similarly, Epac1-ko mice show only a subtle phenotype with increased basal microvascular permeability in skin and other tissues (37). However, it is well established that Epac1 plays a key role in stabilizing the endothelial barrier by regulation of Rho family GTPases (28, 31, 38). Thus, we isolated keratinocytes from Epac1-ko mice to further analyze the role of Epac1 for desmosomal adhesion and cAMP-mediated rescue from PV-IgG-induced loss of adhesion. Epac1-ko keratinocytes showed increased protein levels of Dsg1 and Dsg3 accompanied with elevated Dsg1 mRNA level and enhanced intercellular adhesion. In accordance, in endothelial Epac1-ko cells, VE-Cadherin, the cadherin required for endothelial barrier integrity and for cell junction formation, was upregulated also (31). In addition, similar to our observations in keratinocytes, Epac2 expression was upregulated in Epac1-ko endothelial cells when compared with WT cells, which may serve as a rescue mechanism. Upregulation of desmosomal proteins to overcome compromised adhesion was shown to act as a compensatory mechanism. For instance, Dsg2 is upregulated in Dsg3-deficient cells (39, 40). Differences between isolated keratinocytes and whole skin might be explained by different mechanisms to compensate Epac1 deficiency in vivo and in vitro.

In addition, binding frequencies of Dsg1 and Dsg3 single molecule interactions measured by AFM were elevated in Epac-ko cells, suggesting that enhanced Dsg1 and Dsg3 levels contribute to improved adhesion. In line with this, in plakophilin-ko cells, decreased Dsg3 binding frequencies and membrane availability were accompanied by loss of intercellular adhesion (32). Moreover, Epac1-ko keratinocytes depicted more Dsg3 binding events on cell borders than WT cells, where events were equally distributed between cell surface and border, as shown before (41). Reorganization of binding events was reported to correlate with the adhesive functions. For instance, cAMP induced a shift of Dsg2-dependent binding events toward cell borders in cardiomyocytes accompanied with strengthening of desmosomal adhesion (20). Vice versa, anti-Dsg1 pemphigus autoantibodies were shown to cause a p38MAPK- and keratin-dependent redistribution of Dsg1-dependent binding events away from sites of cell-cell contact in addition to loss of intercellular adhesion (33). Redistribution of Dsg3 binding events in Epac-ko cells, therefore, may also involve keratins or keratin-dependent signaling, as reported before (40). In summary, our data demonstrate higher Dsg1 and Dsg3 protein levels in Epac1-ko cells and enhanced Dsg1 and Dsg3 binding frequencies that strengthen keratinocyte adhesion.

### Epac1 contributes to the protective effect of apremilast on PV-IgG–induced loss of keratinocyte adhesion.

cAMP serves as a rescue mechanism and its pharmacological elevation stabilizes keratinocyte cohesion in PV (19, 34). The second major cAMP-effector protein, Epac1, is important for cAMP-induced, integrin-mediated cell adhesion as well as E-Cadherin–mediated intercellular adhesion (29, 42). Here, we show that the protective effect of apremilast on pemphigus antibody-induced loss of keratinocyte adhesion is Epac1 dependent in vitro and in vivo. Previously, we also reported an involvement of PKA, indicating that both downstream signaling pathways contribute to protective cAMP signaling in pemphigus (19). Surprisingly, a combination of the adenylate cyclase activator forskolin and the PDE4 inhibitor rolipram (F/R) was protective in WT as well as in Epac1-ko keratinocytes, indicating that higher cAMP levels may compensate for missing Epac1. In addition, it is possible that the diverse functions of cAMP are regulated differentially in a spatiotemporal manner dependent on where, when, and how much cAMP levels are enhanced by apremilast or F/R (43, 44).

Taken together, our study demonstrates that Epac1, together with PKA, is involved in the mechanism underlying the apremilast-mediated protection from PV-IgG–induced loss of adhesion.

### Apremilast induces phosphorylation of Pg and Rap1 activation downstream of Epac1.

Recently, we observed that the protective effect of apremilast in pemphigus was associated with Pg phosphorylation (19). In line, enhanced cardiomyocyte cohesion in response to cAMP was paralleled by PKA-dependent phosphorylation of Pg at S665 (20). Here, we show a role of Epac1 in this mechanism, suggesting Epac1 to be required for apremilast-induced phosphorylation of Pg at S665, as outlined above. This was different to F/R-induced phosphorylation of Pg at S665, which was independent of Epac1 and in line with the keratinocyte dissociation assay, where F/R, in contrast to apremilast, still rescues from pemphigus antibody-induced loss of adhesion in Epac1-ko cells. Taken together, these data underline the crucial role of Pg phosphorylation at S665 for protective cAMP signaling in pemphigus.

Keratinocytes display protective mechanisms, including hyperadhesion, Dsg2 upregulation, and cAMP to strengthen adhesion (34, 39, 45). Recently, we reported that apremilast-mediated cAMP increase strengthens keratin network and thereby stabilizes desmosomal adhesion (19). Keratins are coupled to the desmosomal plaque via the plaque protein Dp. Phosphorylation of Dp at S2849 by PKC negatively regulates Dp-keratin interaction and therefore is important for fine tuning of desmosomal adhesion to environmental changes, such as wound healing (28, 35). Here, we show that apremilast did not modulate Dp phosphorylation at S2849 or S165, indicating that phosphorylation of Pg and Dp is independent from each other and Dp phosphorylation is not crucial for the protective effects of cAMP signaling on desmosome adhesion.

Finally, apremilast induced Rap1 activation downstream of Epac1. Todorovic et al. published a mechanism where cAMP induced desmosome assembly and desmosome stabilization via Epac1/Rap1 and Plakophilin 3 (30), which could account for strengthened keratinocyte adhesion. Similarly, in endothelial cells, cAMP stabilizes adherens junctions via Epac1/Rap1 signaling, which induces reorganization of the actin network and thereby stabilizes VE-Cadherin adhesion (46). A comparable mechanism could be present in keratinocytes where cAMP strengthens keratin anchorage and thereby desmosomal adhesion. Our previous finding of enhanced Pg-mediated cytoskeletal anchorage of Dsg3 after treatment with apremilast supports this theory (19).

Taken together, our data show a compensatory upregulation of desmosomal cadherins together with Epac2, which suggests a requirement of Epac1 for keratinocyte cohesion under resting conditions. More importantly the data provide evidence for a role of Epac1 in cAMP-mediated stabilization of keratinocyte adhesion. The mechanism involves Epac1- and PKA-dependent phosphorylation of Pg and potentially other mechanisms, such as Rap1 activation.

## Methods

### Sex as a biological variable.

Our study examined male and female animals and similar findings are reported for both sexes.

### Mice.

WT and Epac1-ko mice used in this study were obtained from Prof. Stein Ove Døskeland (Department of Biomedicine, University of Bergen, Bergen, Norway) (37). Mice were maintained in breeding facility (Department of Psychiatry and Psychotherapy, Ludwig-Maximilians-Universität München, Munich, Germany) under approved animal breeding proposal of an ethical board of the Regierung von Oberbayern (Vet_02-19-172). The mice were maintained in cages of the IVC System “Seal-Safe” (Tecniplast) at 22 ± 1.5°C and a humidity 50 ± 5% with a 12 hour/12 hour dark/light cycle.

### Neonatal pemphigus mouse model.

The pemphigus mouse model was established using 2-day-old WT and Epac1-ko mice following an already published protocol (19). Animal experiments were approved by an ethical board of Regierung von Oberbayern (Vet 02_21_205). Briefly, neonatal mice were maintained at 37°C, fed every 2 hours, and monitored using a score sheet. Subepidermal injections were performed in the back skin of the mice either with 50 μL of vehicle or apremilast (1 μM) for 2 hours. Then, mice were injected subepidermal with 50 μL ctr-IgG (of healthy volunteers) or PV-IgG (of a patient with pemphigus vulgaris, immunopheresis material, concentration: 35.3 mg/mL). After 10 hours, mice were sacrificed by decapitation, a defined stress was applied by pinching (similar to Nikolsky sign), and skin samples of the area of injection were embedded in Tissue-tec (Leica).

### Histology.

For histological analysis, cryosectioning with 7 μm–thick serial sections was performed using a cryostat microtome (HM 500 OM MICROM International GmbH). The entire sample was sliced and screened for blisters. H&E staining of representative slices was accomplished according to standard procedures and mounted in DEPX (Sigma-Aldrich). Stained sections were imaged using a slide scanner (Mirax MIDI, Zeiss) equipped with a plan apochromatic objective (× 20), and the resulting images were analyzed using Case Viewer (3DHistech).

### Cell culture and generation of murine keratinocytes.

Experiments were performed in immortalized murine keratinocytes isolated from Epac1-ko and WT mice according to a well-established protocol (39, 47). Briefly, epidermis was obtained from neonatal mice by incubation of the skin with Dispase II (Sigma-Aldrich), and epidermal cells were isolated using Accutase (Sigma-Aldrich), seeded in complete FAD media (0.05 mM CaCl_2_, PAN Biotech) on collagen I–coated (rat tail; BD Bioscience) flasks and maintained at 35°C and 5% CO_2_. After 10–15 passages, keratinocytes immortalize spontaneously. Differentiation was achieved by adding high Ca^2+^ (1.2 mM) to the medium 48 hours before the experiments.

### Immunostaining.

For immunostaining of murine epidermis, slices were heated at 60°C (45 minutes), fixed with 2% paraformaldehyde (15 minutes), and permeabilized with 1% Triton X-100 (45 minutes). Cells were fixed with ethanol (30 minutes) and aceton (3 minutes).

After blocking with 3% BSA and 1% normal goat serum (1 hour) samples were incubated with primary antibodies listed in Supplemental Table 1 overnight at 4°C. After washing alexa488- or Cy3-coupled goat-anti-rabbit or goat-anti-mouse secondary antibodies (dilution 1:600, all from Dianova) (1 hour) and 4′,6-diamidin-2-phenylindol (DAPI, 1 mg/mL) for staining of nuclei were used (10 minutes). Next, samples were mounted with NPG (1% n-Propylgallat and 60% Glycerin in PBS), and images were acquired with a Leica SP5 confocal microscope equipped with a 63× oil objective (Leica)

### AK23 and purification of patient IgG fractions and AFM proteins.

AK23, a pathogenic monoclonal anti-Dsg3 antibody derived from a PV mouse model (MBL), was used at a concentration of 75 μg/mL.

Sera samples of healthy human volunteers and patients with PV were used with informed and written consent under approval of the local ethic committee of University of Lübeck (AZ12-178) and University of Würzburg (AZ159/06). Patients with PV were diagnosed based on national and international criteria (48, 49), and antibody profiles were measured using Dsg1- and Dsg3-ELISA (Euroimmun or MBL). Resulting titers are given in Table 1. IgG-fractions were purified using protein A affinity chromatography (Thermo Fisher Scientific), as described previously (6, 19).

Dsg1- and Dsg3-Fc constructs, containing the full extracellular domain of the respective protein and a human Fc-tag, were expressed in Chinese hamster ovary cells. Recombinant proteins were purified from supernatants using protein A agarose affinity chromatography (Thermo Fisher Scientific), as described before (6, 19). Purity of the proteins was tested by Coomassie staining and Western blot analysis.

### Keratinocyte dissociation assay.

After preincubation with respective mediators (100 μM apremilast [Biomol], 1 μM Esi-09, 5 μM forskolin, 10 μM rolipram [all 3 obtained from Sigma-Aldrich, Taufkirchen, Germany]) for 1 hour, AK23 or PV-IgG were added for 24 hours. The confluent keratinocyte monolayers were detached from the well bottom using Dispase II (Sigma-Aldrich) and Collagenase I/II (Thermo Fisher Scientific). By adding Hanks’ Balanced Salt Solution, the monolayer detachment was terminated and a defined shear stress was applied using an electrical pipette. Resulting fragments were counted and represent an inverse measure for keratinocyte cohesion.

### Cell and tissue lysis.

Skin from neonatal mice or cells were lysed using SDS lysis buffer (25 mM HEPES, 2 mMol EDTA, 25 mM NaF and 1 % sodium dodecyl sulfate, pH 7.6, protease inhibitors). Tissues were homogenized using a Potter-Elvehjem hand homogenizer, and lysates were cleared by centrifugation (14,500 ×*g*, 5 minutes). For some experiments Triton fractionation was performed. Cells were incubated with Triton buffer (0.5% Triton-X-100, 50 mM MES, 25 mM EGTA, 5 mM MgCl_2_, pH = 6.8, protease inhibitors) on ice (20 minutes), scraped, and afterward centrifugated (14,500 ×*g*, 5 minutes) to separate the Triton-soluble, noncytoskeletal-bound from the Triton-nonsoluble, cytoskeletal-bound proteins. Supernatants were collected, and pellets containing the Triton-insoluble, desmosome-containing fraction were resuspended in SDS lysis buffer and sonicated. Protein amount was determined with a Pierce BCA protein assay kit (Thermo Fisher Scientific).

### Rap1 pulldown.

Rap1 GTPase activation was measured using the Active Rap1 Detection Kit (Cell Signaling) following the manufacturer’s instructions. Briefly, activated Rap1-GTP was pulled down using GST-RalGDS-RBD. Subsequently, equal amounts of protein from control and treated cells were loaded for Western Blot analysis.

### Western blot analysis.

Electrophoresis and Western blotting were performed using standard protocols. Primary antibodies (overnight, 4°C, Supplemental Table 1) in 5% BSA, Tris-buffered saline with Tween 20, and HRP-coupled secondary antibodies (1 hour) were added. Blots were developed with a Western blot developer Amersham Imager 600 (Thermo Fisher Scientific).

### Atomic force microscopy measurements.

For Atomic force microscopy (AFM) measurements on living murine keratinocytes, a well-established protocol with a NanoWizard 3 AFM (JPK-Instruments) mounted on an inverted optical microscope (Carl Zeiss) was used, which allows selection of the scanning area by visualizing the cells with a 63 × objective. Pyramidal-shaped D Tips of Si3N4 MLCT cantilevers (Bruker) with a nominal spring constant of 0.03 N/m and tip radius of 20 nm were functionalized with purified Dsg3-Fc or Dsg1-Fc, containing the full extracellular domains of Dsg3 or Dsg1 (concentration: 0.15 mg/mL), as described previously (50). Here, a flexible heterobifunctional acetal-polyethylenglycol (PEG, BroadPharm) was interspaced between the tip and the recombinant Dsg-Fc. AFM was used either in the Quantitative Imaging (QI, for overview images) or the Force Mapping (FM, for force measurements) mode. In the QI mode, an area of 60 × 40 μm was sampled with setpoint of 0.5 nN and a pulling speed of 50 μm/s and a Z-length of 2 μm. These settings were applied to avoid cell alterations due to mechanical stress and allowed to identify areas of cell-cell contact, further referred to as cell borders. In the FM mode, each map consisted of distinct force-distance cycles, with each cycle representing 1 pixel of the map covering an area of 1.25 × 6 μm along a cell border. Settings were adapted to setpoint 0.5 nN, pulling speed 10 μm/s, Z-length 2 μm, and a resting contact time of 0.1 s. JPK NanoWizard was used for data analysis (JPK Instruments). To calculate the distribution coefficient, binding frequencies along the elevated cell borders (marked with dotted lines in Figure 2 and Supplemental Figure2) were divided by binding frequencies at the cell surface areas surrounding the cell borders, as described in detail previously (33).

### PCR.

PCR analysis was used to determine the mRNA presence and abundance of certain targets of interest (Supplemental Table 2). For that purpose, cDNA was amplified/synthesized from equal amount of total RNA using SuperScript II Reverse Transcriptase (Thermo Fisher Scientific). Total RNA extraction from Epac1-ko cells and a respective control cell line was accomplished with the support of RNeasy Plus Mini Kit (Qiagen), following the manufacturer’s instructions. For an amplification of the specific targets, a PCR master mix that contains KAPA polymerase, dNTPs, a buffer system with a loading dye for electrophoresis, specific primer pairs, and equal amounts of cDNA as a template was assembled/prepared, and PCR with the following cycling conditions was run: 3 minutes of initial denaturation at 95°C, 35 cycles of 15 seconds denaturation at 95°C, 15 seconds primer annealing at 57°/60°C, followed by 20 seconds of extension at 72°C. GAPDH was used to verify equal loading of the samples on an agarose gel, prestained with HDGreen Plus DNA stain (INTAS Science imaging). Imaging of the gels was accomplished with iBright 1500 instrument (Invitrogen). Evaluation of the pixel intensity of the detected amplicons was performed with ImageJ.

### Image processing.

Images were processed with Photoshop CS5 (Adobe) and LAS X life science (Leica). Image J (NIH) was used to quantify fluorescence intensity (fluorescence staining) and band intensity (Western blot- and PCR analysis). Case viewer (3DHISTECH) was used for analysis of blister length (H & E staining). AFM data were analyzed with JPK Data Processing software (JPK-Instruments), and peak fit analysis to determine most probable unbinding forces was performed using the Extreme fit function of Origin Pro 2016, 93G (OriginLab).

### Statistics.

For statistics and graphs, GraphPad Prism (GraphPad Software) was used. To compare means of more than 2 samples to respective controls, 1-way or 2-way ANOVA with corrections as indicated were used and significance was determined for a *P* value of < 0.5. For 2 independent groups, an unpaired 2-tailed *t*-test was applied.

### Study approval.

Mouse maintenance was approved by an animal breeding proposal of an ethical board of the Regierung von Oberbayern (Vet_02-19-172). Animal experiments were approved by an ethical board of Regierung von Oberbayern (Vet 02_21_205).

### Data availability.

The datasets generated during the current study are presented in the main manuscript or as Supplemental material. Values for all data points in graphs are reported in the Supporting Data Values file. Further information is available from the corresponding authors on reasonable request.

## Author contributions

AMS, JW, and FV designed research study. AMS, FCB, DK, EB, SM, MECS, AKEH, MYR, SE, DTE, and FV conducted experiments and acquired and analyzed data. MG and ES provided PV-IgG. AMS, JW and FV supervised MD students (FCB, MECS, SE). AMS wrote and prepared original draft. All other authors contributed to reviewing and editing of the manuscript. All authors read and approved the final manuscript.

## Supplementary Material

Supplemental data

Supporting data values

## Figures and Tables

**Table 1:**
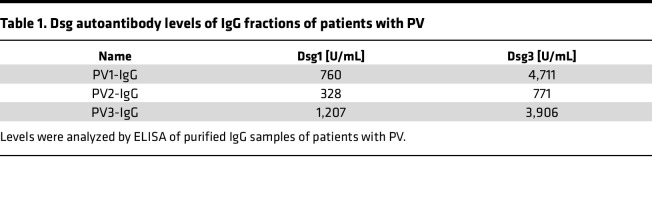
Dsg autoantibody levels of IgG fractions of patients with PV

## Version 1 04/29/2025

In-Press Preview

## Version 2 05/22/2025

Electronic publication
