# Supplementary material for: Epac1 contributes to apremilast-mediated rescue of pemphigus autoantibody-induced loss of keratinocyte adhesion
Source: JCI Insight. 2025 Apr 29;10(10):e187481. doi: 10.1172/jci.insight.187481 (PMC12128971; doi:10.1172/jci.insight.187481)
Supplement: Supplemental data [file jciinsight-10-187481-s234.pdf]

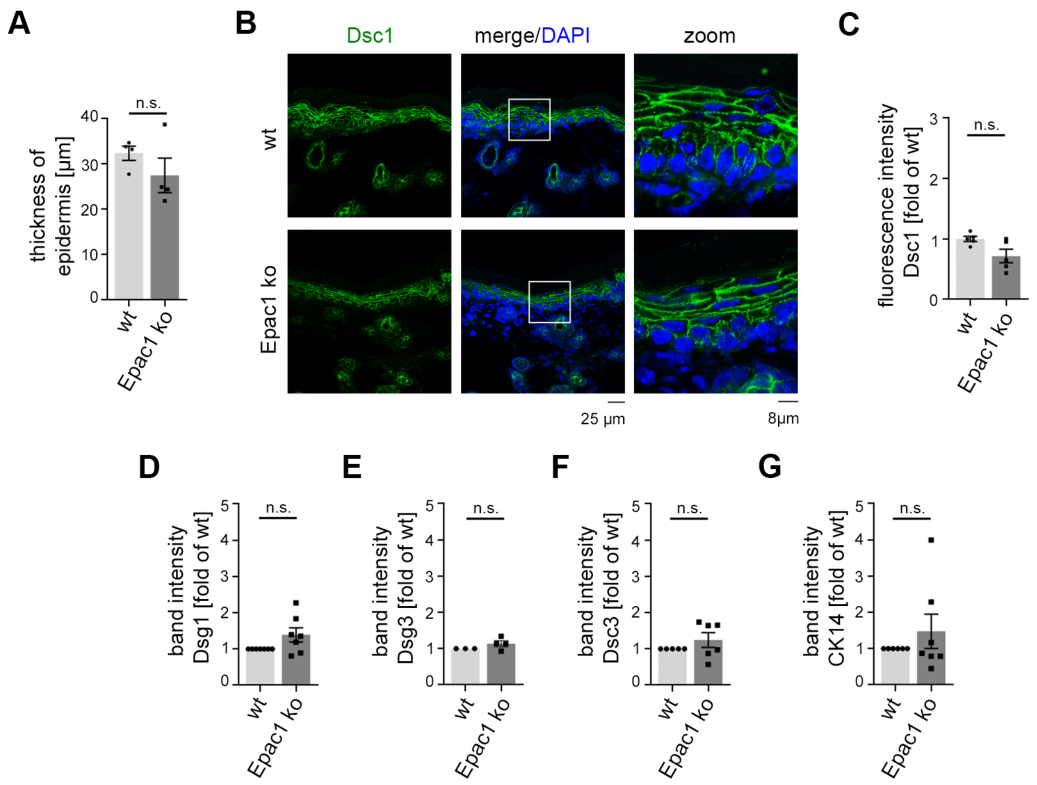

Fig. S1: Epac1 ko epidermis shows no alterations.

A Quantification of thickness of epidermis depicted in Fig.1 (n=4). Fluorescence staining (**B**) and quantification (**C**) of Dsc1 in murine epidermis showed no difference between wt and Epac1 mice (representative of n>4). (**D-G**) Quantifications of Western blots shown in Fig.2. Bars indicate mean value ±SEM. *P < 0.05. Two-tailed Student's t test.

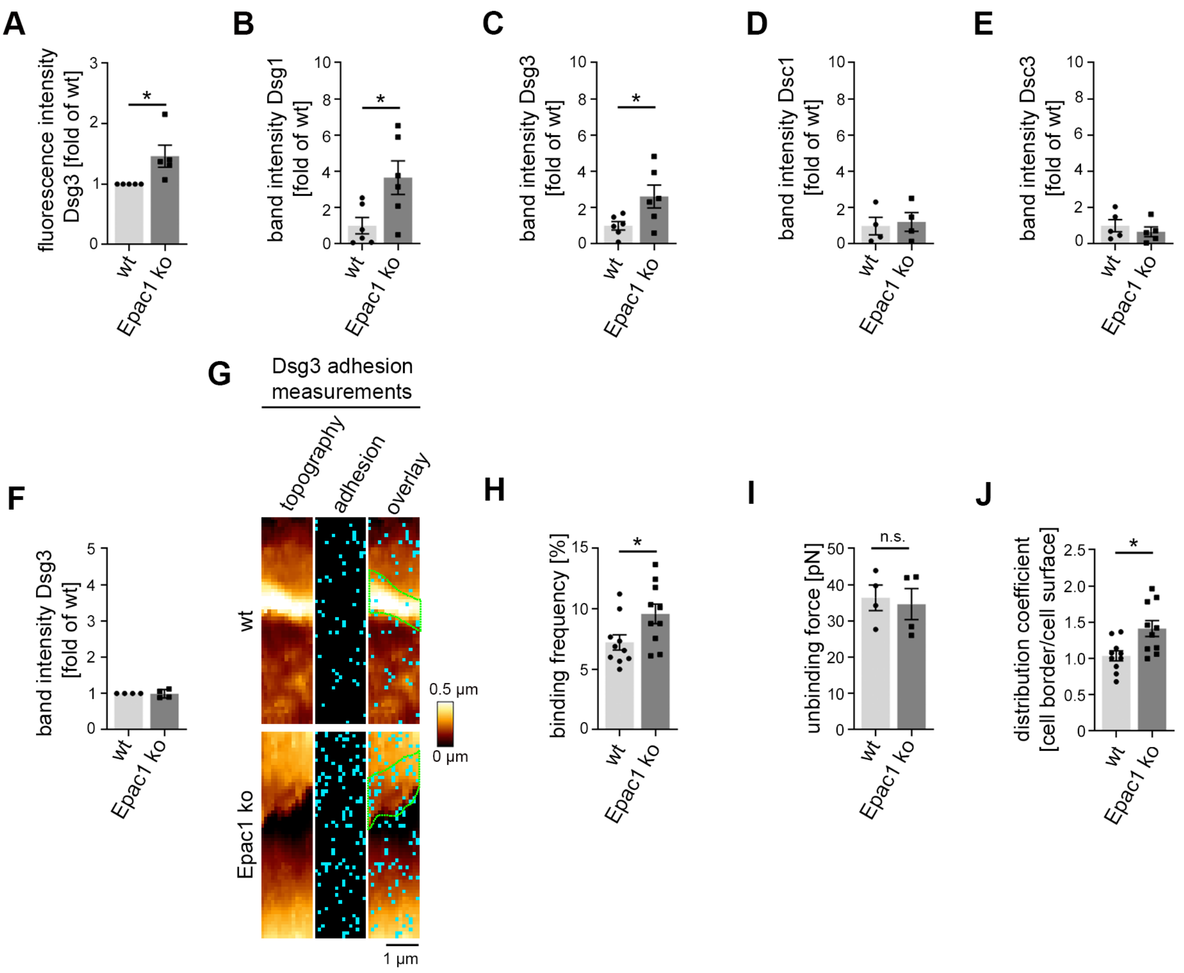

Fig. S2: Desmosomal proteins are upregulated in Epac1 ko keratinocytes.

Quantification of fluorescence intensities of Dsg3 immunofluorescence (**A**) and band intensities of Western blots of Triton insoluble fractions of wt and Epac1 ko keratinocytes depicted in Fig.1 (**B-E**). **F** Quantification of band intensity of Dsg3 PCR, normalized to the loading control GAPDH. **G** Topography and adhesion images of atomic force microscopy (AFM) measurements on living keratinocytes using a Dsg3-functionalized tips. Each pixel represents a force-distance-curve. In the adhesion panel each blue pixel represents a Dsg3-specific binding event. **H-J** Quantification of AFM adhesion measurements. Epac1 ko cells displayed higher Dsg3 binding frequencies (**H**) with unaltered unbinding forces (**I**) and enhanced number of binding events at cell borders (**J**) compared to wt cells. Cell borders are marked in green (10 cell borders from 4-5 independent experiments with 900 force-distance curves/ cell border). Bars indicate mean value ±SEM. *P < 0.05. Two-tailed Student's t test.

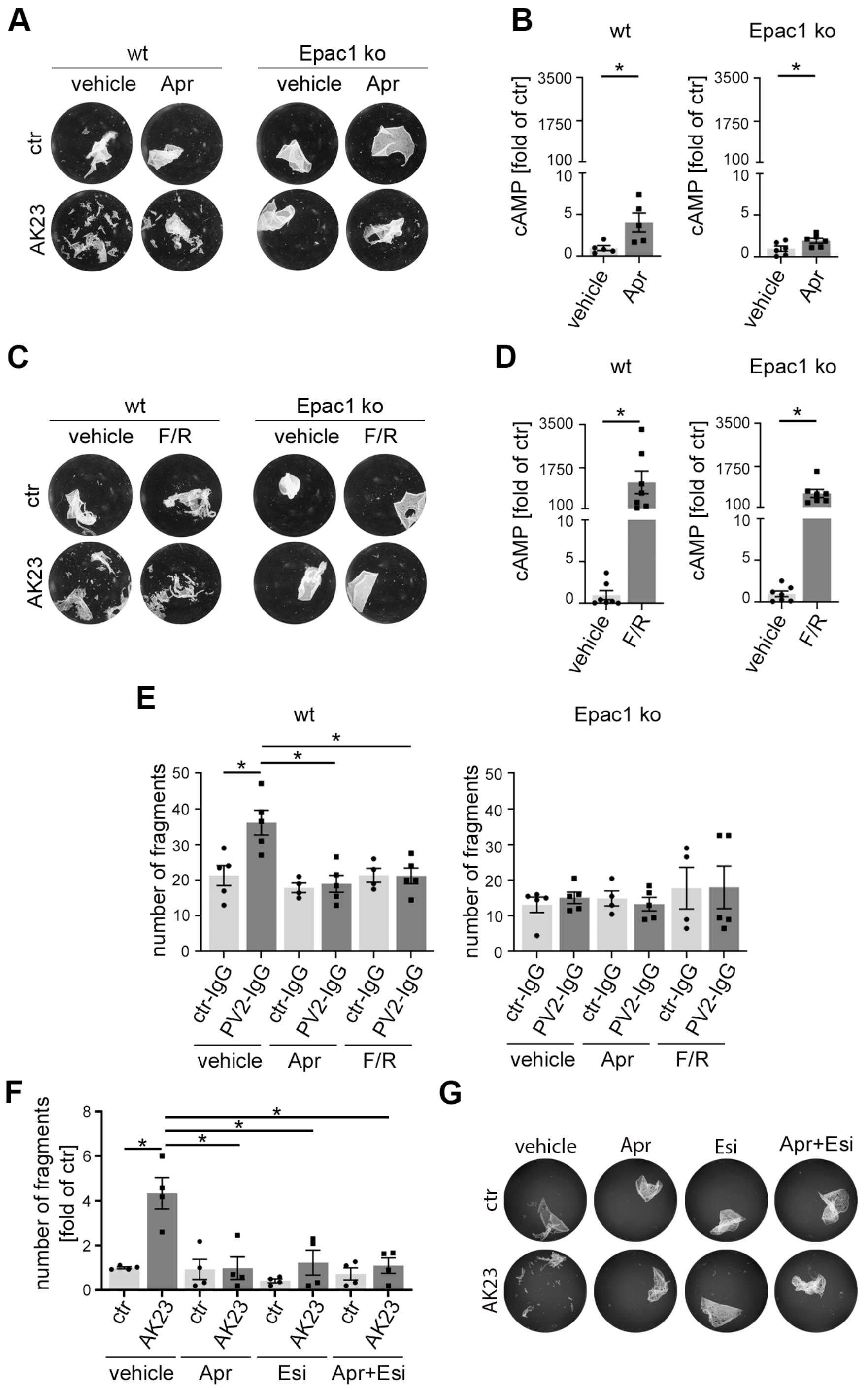

Fig. S3: Apremilast induces cAMP in Epac1 ko keratinocytes and cells with impaired Epac1 showed increased intercellular adhesion.

A, C Representative pictures of keratinocyte assays presented in Fig. 3A. **B, D** cAMP ELISA of wt and Epac1 ko keratinocytes after incubation with apremilast (Apr) or forskolin/rolipram (F/R), respectively (n>5). **E** Keratinocyte dissociation assays of wt or Epac1 ko keratinocytes revealed no loss of adhesion of Epac1 ko cells after treatment with PV2-IgG. Neither Apr nor F/R affected cell adhesion of Epac1 ko cells (n>4). **F** Inhibition of Epac1 by Esi-09 improved intercellular adhesion upon AK23 treatment, which could not be further enhanced by addition of Apr (n=4). **G** Representative pictures of keratinocyte assays of F. Bars indicate mean value ±SEM. *P < 0.05. Two-tailed Student's t test (B, D). Two-way ANOVA with Bonferroni correction (E, F). Pemphigus vulgaris IgG (PV-IgG), IgG of healthy volunteers (ctr-IgG)

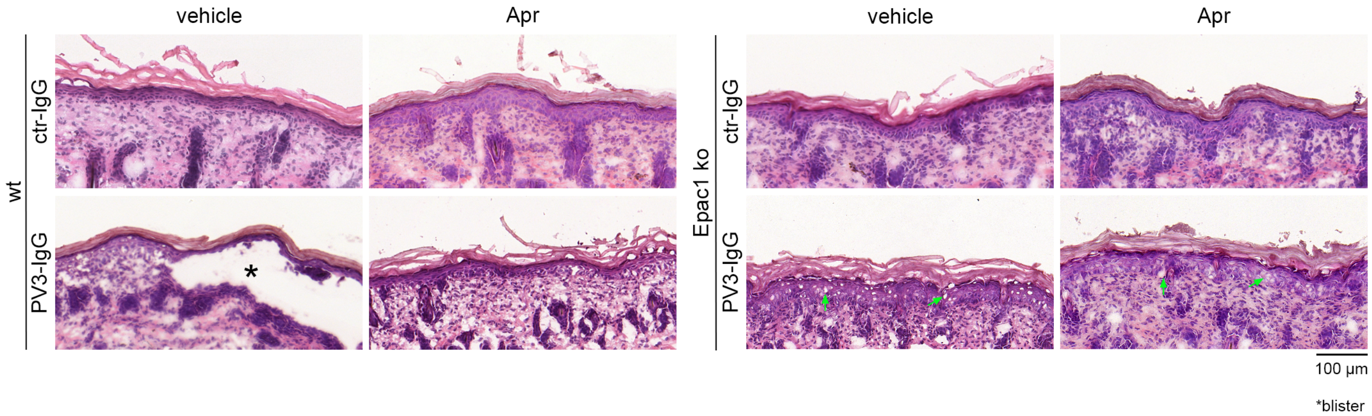

Fig. S4: Apremilast does not further enhance improved cell cohesion of Epac1 ko mice in the pemphigus mouse model.

H.&E.-staining of epidermis of the pemphigus mouse model after injection of vehicle or apremilast (Apr) prior to injection of PV3-IgG or ctr-IgG. PV-IgG induced suprabasal blistering of wt epidermis, which was rescued by apremilast. Micro-blisters in Epac1 ko epidermis (marked with green arrows) were not improved by apremilast (representative of n>3). Pemphigus vulgaris IgG (PV-IgG), IgG of healthy volunteers (ctr-IgG)

Supplemental Table 1: Antibodies

Antibody	Species	Company	Dilution
**anti-Dsg1**	mouse	Santa cruz, Dallas, USA	1:1000 (WB)<br>1:100 (IF)
**anti-Dsg2**	rabbit	Abbexa, Cambridge, UK	1:1000 (WB
**anti-Dsg3**	rabbit	Biozol, Eching, Germany	1:1000 (WB)<br>1:100 (IF)
**anti-Dsg3 (AK18)**	mouse	MBL, Schaumburg, USA	1:1000 (WB)<br>1:100 (IF)
**anti-Dsc1**	rabbit	Abcam, Cambridge, UK	1:1000 (WB)<br>1:100 (IF)
**anti-Dsc3**	rabbit	Progen, Heidelberg, Germany	1:1000 (WB)<br>1:100 (IF)
**anti-Dp**	rabbit	Abclonal, Woburn, USA	1:1000 (WB)<br>1:100 (IF)
**anti-α-Tubulin**	mouse	Abcam, Cambridge, UK	1:1000 (WB)
**anti-GAPDH**	mouse	AviaSysBio, San Diego, USA	1:1000 (WB)
**anti-Epac1**	rabbit	Abbexa, Cambridge, UK	1:1000 (WB)
**anti-P-Dp (S2849)**	rabbit	Gift of K. J. Green, Chicago, USA	1:1000 (WB)
**anti-P-Dp (S165)**	rabbit	Cell signaling, Leiden, Netherlands	1:1000 (WB)
**anti-p-Pg**	mouse	Yeruva, Kempf et al. 2020	1:10 (WB)
**anti-Pg**	mouse	Progen, Heidelberg, Germany	1:1000 (WB)
**anti-CK14**	mouse	Abcam, Cambridge, UK	1:1000 (WB)<br>1:100 (IF)

Yeruva, S., E. Kempf, D. T. Egu, H. Flaswinkel, D. Kugelmann and J. Waschke (2020). "Adrenergic Signaling-Induced Ultrastructural Strengthening of Intercalated Discs via Plakoglobin Is Crucial for Positive Adhesiotropy in Murine Cardiomyocytes." Front Physiol **11**: 430.

Supplemental Table 2: Primers used for PCR analysis

**Target analyzed**	**5'-> 3'**	**Amplicon size (bp)**
**Mouse Dsg1**	FW: ACTGTGTTAAATGTCATCGAGGG<br>REV: TGCCTGTTCTTGAGTCAACAAC	198
**Mouse Dsg3**	FW: CAGCCATAGTTGATCGTGAGG<br>REV: ATCTGCATCCGTGGCATTTAG	221
**Mouse Epac1**	FW: CAGGTCAGCGTACGGATGAAGAAC<br>REV: GCTTCCACATCCTTGATGATGCG	378
**Mouse Epac2**	FW: ATCTACGACGAGCTCCTTCATATTAAA<br>REV: GACTACATTCACGGATCCTTTCAGA	176
**Mouse GAPDH**	FW: GTCATCATCTCCGCCCCTTCTGC<br>REV: GATGCCTGCTTCACCACCTTCTTG	443
**Mouse Dsg3**	FW: GCTGCCTCCTCTGTCAGATATG<br>REV: GGAGGTCTTACCAGTGTTTTCGTC	177
**Mouse Dsg3**	FW: CAGCCATAGTTGATCGTGAGG<br>REV: ATCTGCATCCGTGGCATTTAG	221
